# Social welfare and economic equality: healthcare expenditure as a moderator

**DOI:** 10.3389/fpubh.2025.1547027

**Published:** 2025-04-25

**Authors:** Gai Liu

**Affiliations:** School of Marxism, Yangzhou University, Yangzhou, Jiangsu, China

**Keywords:** social welfare expenditure, economic equality development, healthcare expenditures, moderating effect, free medical care

## Abstract

**Introduction:**

This study aims to examine the impact of social welfare expenditure on household economic equality, as well as the moderating effect of healthcare expenditure on this relationship. Additionally, the study seeks to propose policy recommendations to address universal health issues and enhance the overall level of national welfare.

**Methods:**

Utilizing the CFPS database, this study employs a two-way fixed effects model, along with moderating effect models, to investigate the impacts of social welfare expenditure, household healthcare expenditures, family education expenditures, and household housing expenditures on the advancement of household economic equality.

**Results:**

This study found that for every 1% increase in social welfare expenditure, family economic equality can be improved by 0.033. However, the study also revealed a decrease of 0.069 in the employment quality of the head of the household, indicating that social welfare expenditure negatively impacts this employment quality. Additionally, the moderating effect analysis demonstrated a significantly negative interaction between healthcare expenditure and social welfare expenditure, suggesting that family healthcare expenditure diminishes the positive effect of social welfare expenditure on the advancement of family economic equality.

**Conclusion:**

The government should optimize and expand the level and efficiency of social welfare expenditure. For instance, the government could implement a ‘universal free medical care’ policy. Specific measures may include waiving medical insurance premiums, ensuring full reimbursement, and adopting a ‘treatment first, settlement later’ system. These initiatives will effectively alleviate the economic burden of health-related issues on families and promote equitable development of family economies. Furthermore, the government should also enhance policies related to employment quality. Such measures will contribute to optimizing the level and efficiency of social welfare expenditure, fostering economic equality, and narrowing the gap between the rich and the poor.

## 1 Introduction

Addressing economic inequality and narrowing the gap between the rich and the poor is a significant global issue. Studies have indicated that economic inequality has increased markedly in most countries over recent decades (1, 2). This research employs a substantial dataset and empirical analysis to investigate the potential impact of social welfare expenditures on promoting economic equality, as well as the moderating role of household healthcare expenditures in this relationship. Studies have demonstrated a significant convergence relationship between national health expenditure and economic growth. Specifically, an increase in high-quality national health expenditure leads to enhanced human capital, which in turn fosters value-added production and efficient output, ultimately contributing to GDP growth (3).

Since the reform and opening up, the Chinese economy has achieved remarkable progress, with per capita GDP soaring from 381 yuan in 1978 to 89,424 yuan in 2023, resulting in significant improvements in national strength and living standards for the populace. However, according to the Gini Index published by the World Bank, despite high levels of income inequality in both China and Russia, China’s Gini coefficient has consistently exceeded that of Russia since the late 1990s (4). From 1990 to 2010, China experienced one of the fastest increases in income inequality among the BRICS countries, as evidenced by a rise in its Gini coefficient from 32.7 to 47.8 (5). Furthermore, according to the latest data from the National Bureau of Statistics, the per capita disposable income of national residents in 2023 is projected to be 39,218 yuan. This figure includes a per capita disposable income of 51,821 yuan for urban residents and 21,691 yuan for rural residents (6), highlighting an income gap between urban and rural areas that exceeds 2.3 times. Analysis of the five income groups in China reveals that the per capita disposable income for the low-income group is 9,215 yuan, the lower middle-income group is 20,442 yuan, the middle-income group is 32,195 yuan, the upper middle-income group is 50,220 yuan, and the high-income group is 95,055 yuan (6). Notably, the high-income group earns 10.3 times more than the low-income group. This widening income disparity not only impedes equitable economic development but also presents challenges to social equity and stability. Research indicates that societies with robust social welfare initiatives tend to achieve higher scores on various economic equality indices (7), thereby better managing the divide between the affluent and the impoverished (8). For instance, effective welfare programs can alleviate poverty (9, 10) and enhance access to essential services such as healthcare and education (11–14). Consequently, the challenge facing China’s future development has shifted from a focus solely on social welfare security to a broader concern regarding the economic equality of families. However, while the direct relationship between social welfare expenditure and equitable economic development (the unequal relationship between politics and the economy) may appear logical, it remains relatively underexplored in current research (15). In this context, our study holds significant value.

In addition, due to the inadequacies and inequities in social welfare policies and systems (16), various challenges have emerged in healthcare, employment, housing, education, and older adults care. These challenges remain largely unresolved (17–19). In the context of a rapidly aging population, healthcare issues have become a critical concern for families across the nation. Notably, universal free healthcare has yet to be realized in China. The cost of basic medical insurance for urban and rural residents has escalated from 10 yuan in 2003 to 400 yuan in 2024, marking an increase of nearly 40 times over the years. This surge has become a burden for rural families, particularly those with 4–5 members and no fixed income, leading some to withdraw from the basic medical insurance program (20). Ironically, despite many individuals paying these premiums, the reimbursement rate for medical expenses remains limited, resulting in substantial inequality (21, 22). Families facing catastrophic illnesses often find themselves incurring significant out-of-pocket expenses to continue medical treatment (23, 24). Consequently, many families experience heightened economic pressure due to major or chronic diseases, contributing to the phenomenon of poverty induced by illness (25–27). Some scholars argue that enhancing the reimbursement rate of basic medical insurance and broadening its coverage can promote income equity among older adults households (28). Furthermore, increasing investments in rural pension schemes and medical facilities, along with significant rises in rural spending and reductions in out-of-pocket medical expenses, would not only help mitigate rural–urban disparities but could also contribute to decreased mortality rates among the older adults in rural areas (29).

Current literature predominantly emphasizes macro-level aspects of equitable economic development, such as overall economic growth and macro-level equality indicators like the Gini coefficient. However, it often overlooks critical dimensions, including economic development within households, income disparity, exploitation, and equitable opportunities for development. This macro-focused research methodology may inadequately capture the effects of social welfare expenditure on family economic equality. Furthermore, the application of diverse measurement scales across studies can result in inconsistent findings and conclusions. Although existing literature offers valuable insights into the relationship between social welfare expenditure and equitable economic development, further investigation is necessary to address the identified gaps, particularly within the Chinese context. This study aims to explore the multi-dimensional aspects of social welfare expenditure, considering the employment quality of household heads as a mediating factor influencing economic equality development. Additionally, family healthcare expenditure, family education expenditure, and family housing expenditure will be examined as moderating variables in the relationship between social welfare expenditure and economic equality development. By employing a standardized scientific measurement scale, this study seeks to reduce inconsistencies in research outcomes and provide reliable data for decision-makers. This approach will enhance our understanding of the impact of social welfare expenditure on economic equity development, which is especially crucial given the pressing need to improve both social welfare expenditure and economic equity.

**Figure 1:**
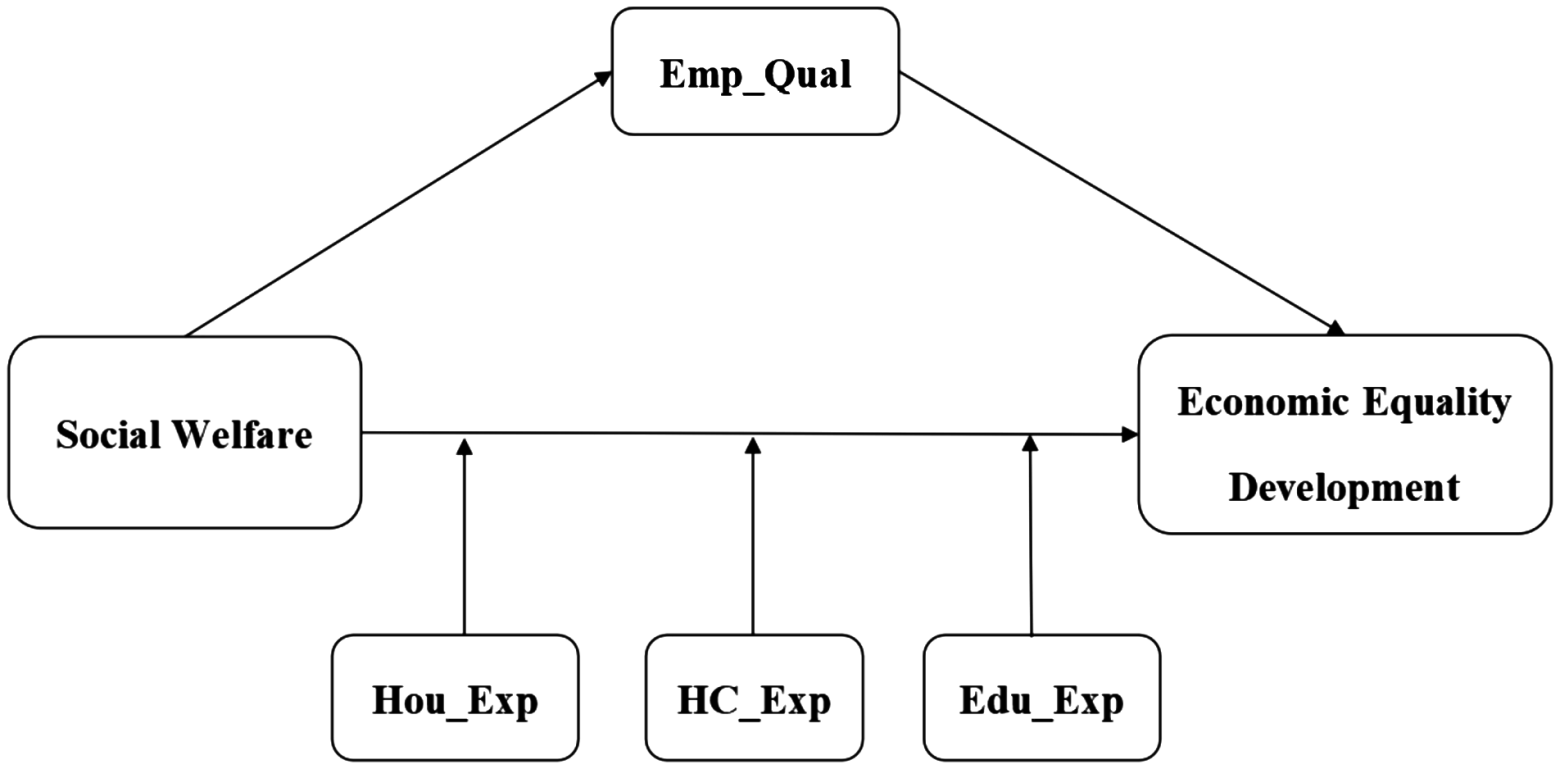
Graphical representation of conceptual framework.

The purpose of this study is to investigate the relationship between social welfare expenditure and the development of household economic equality by analyzing data from the China Family Tracking Survey (CFPS) conducted between 2016 and 2020, utilizing a two-way household panel survey model. This approach enhances our understanding of how social welfare expenditure influences the development of economic equality, allowing for the exploration of potential variations across different economic contexts. The study aims to address three primary questions: first, whether social welfare expenditure directly impacts the development of family economic equality; second, whether social welfare expenditure indirectly influences economic equality development by affecting the employment quality of household heads; and third, whether significant household expenditures (such as those for healthcare, education, and housing) can moderate the relationship between social welfare expenditures and economic equitable development. Through a comprehensive analytical framework (illustrated in Figure 1), this study will examine both mediating and moderating effects, exploring the mechanisms by which social welfare expenditure impacts economic equality development, drawing insights from microeconomics, political science, and sociology. The ultimate goal is to provide decision-makers with empirical evidence to enhance their understanding of how social welfare policies affect economic equity development, enabling the formulation of more effective strategies to optimize social welfare expenditure policies and improve the outcomes of economic equity for families. The extensive use of CFPS data ensures the reliability of the research findings and the practicality of the policy recommendations.

Based on the preceding analysis, this article proposes the following hypotheses:

*Hypothesis* 1: Social welfare expenditure can significantly enhance the development of economic equality among families.

*Hypothesis* 2: Social welfare expenditure influences the level of economic equality development within families by impacting the employment quality of the household head.

*Hypothesis* 3: Major household expenditures (such as healthcare, education, and housing) may moderate the relationship between social welfare expenditures and the development of economic equality within families.

## 2 Materials and methods

### 2.1 Data source and sample

**Figure 2:**
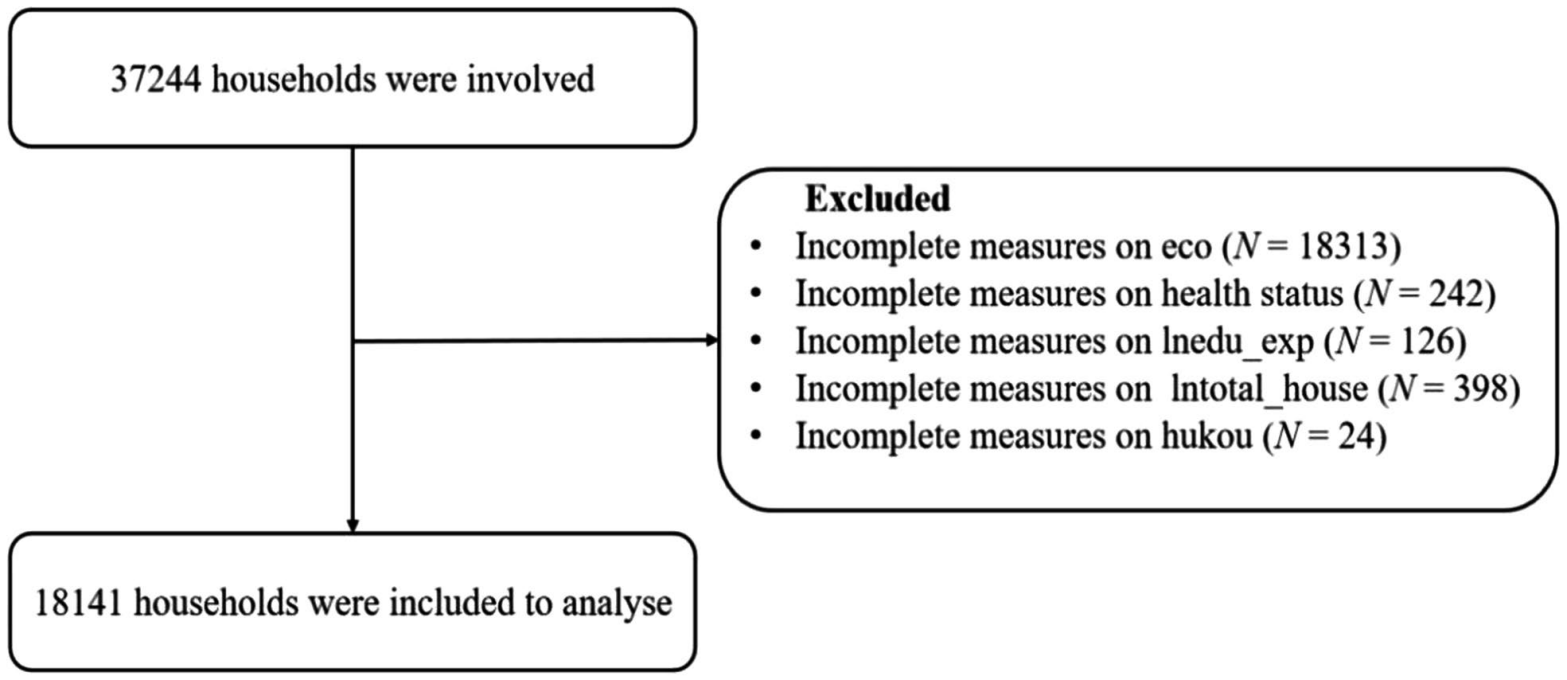
Flowchart of data processing and analysis.

The macro data presented in this paper are sourced from the official website of the National Bureau of Statistics, representing national-level information. In contrast, the micro database is derived from the CFPS (China Household Tracking Survey) database. Together, these macro and micro datasets provide a comprehensive foundation for analyzing the impact of social welfare expenditure on economic equality among households. The national macro database outlines the types and amounts of social welfare expenditures over various years. Meanwhile, the CFPS database, initiated in 2010 by the Institute of Social Science Surveys (ISSS) at Peking University, serves as a nationally representative longitudinal survey encompassing Chinese communities, families, and individuals. This household microdatabase offers detailed insights into the economic structure of households, including types and sources of income, household assets and liabilities, and essential needs such as food, education, healthcare, and housing. Additionally, it includes various demographic details, such as family members’ health status, marital status, education, and employment (30). By controlling for potential confounding factors, we can more accurately estimate the impact of social welfare expenditure on the development of household economic equality. This study utilizes the macro and micro databases from 2016, 2018, and 2020 to clean the original CFPS data, addressing issues of data omissions and outliers to ensure the integrity and accuracy of the dataset. Ultimately, the effective sample size comprises 18,141 households, as illustrated in Figure 2.

### 2.2 Measurements

#### 2.2.1 Economic equality development

The variable examined in this paper is economic equal development. Within the framework of economic equal development, the concepts of “economic development” and “economic equality” are closely intertwined. Accordingly, this paper evaluates and constructs the development level of family economic equality by considering both “economic development” and “economic equality, “guided by the principles of operability, simplicity, and practicality, as referenced in existing literature (28, 31). The specific data utilized for this analysis are derived from the Chinese Family Panel Studies (CFPS), as detailed in Table 1. The calculation method for family discretionary income is defined as the total net family income minus expenses for food, clothing, household goods, housing, cultural and educational entertainment, medical care, transportation, and communication. This definition significantly differs from the concept of family disposable income as defined by official sources. The use of the family discretionary income indicator in this article addresses the issue that national standards may not be applicable due to variations in price levels and consumption expenditure structures across different regions (32), thus providing a more accurate reflection of the net income status of families in various areas. This allows for a more accurate representation of the net income status of households across different regions. In this paper, the entropy weight method is employed to ascertain the degree of economic equality development, denoted as the detailed calculation formulas and methodological steps can be found in the Appendix, specifically in Equations (1–6).

**Table 1:** Economic equality development level indicator system.

First level index	Second level index	Third level index	Impact
Economic development	Income level	Household discretionary income	+
		Household net worth	+
	Living ability	Life satisfaction	+
		Years of schooling	+
Economic equality	Income gap	Kakwani index of discretionary household income	−
		Kakwani index of household net worth	−
	Life gap	If the gap between urban and rural life satisfaction in the city where the family resides is less than the national median, it is recorded as 1, otherwise it is 0	−
		If the gap between urban and rural years of schooling in the city where the family is located is less than the national median, it is 1, otherwise it is 0	−

+ means positive indicator.

#### 2.2.2 Social welfare expenditures

This paper focuses on government social welfare expenditure as the primary explanatory variable, grounded in existing theoretical analysis. According to the official statements of the Chinese government and the indicators outlined in the “14th Five-Year Plan” related to people’s livelihood and welfare, the scope of government social welfare expenditures in this study encompasses education, medical care, housing, social security, and employment (33). The specific data utilized in this analysis are sourced from the official website of the National Bureau of Statistics (National Data Network). The level of government social welfare expenditure is denoted by the term “social welfare.”

#### 2.2.3 Employment quality of household heads

According to the theoretical analysis presented above, government social welfare expenditure may indirectly influence the economic equality development level of households by affecting the employment quality of household heads (34–37). Therefore, employment quality is designated as an intermediate variable in this study. As a comprehensive concept, employment quality is measured using the method developed by Leseschke and Watt, which constructs a multi-dimensional job quality index (38). This approach has also been adopted by numerous domestic scholars to assess employment quality (39, 40). Specific indicators include the income dimension (monthly wage income), the labor supply dimension (weekly working hours), the job stability dimension (whether a labor contract is signed), and the job security dimension (whether medical or pension insurance is provided). Considering the characteristics of the sample data and the actual situation, this paper utilizes the first three indicators to construct the employment quality index. The construction method is as follows: First, each dimension index is standardized; secondly, to ensure that the changes in index values and the final employment quality index align in an economically meaningful direction, the inverse of weekly working hours is reversed. Finally, an equal weight weighted average of the standardized variables for each dimension is computed to obtain the employment quality index (40). The employment quality of the head of the household is denoted by the term “Emp_Qual.” The mechanism through which the intermediary variable impacts this relationship is illustrated in Figure 1.

#### 2.2.4 Moderating variables

This paper identifies three primary moderating variables, supported by relevant research literature: healthcare expenditure (HC_Exp), education expenditure (Edu_Exp), and housing expenditure (Hou_Exp) (41). Daily expenditures for Chinese families encompass various categories, including food, clothing, household equipment, daily necessities, housing, cultural activities, education, entertainment, healthcare, transportation, communication, and other expenses. Among these categories, healthcare, education, and housing expenses constitute a significant portion of a family’s budget, particularly for those with ill family members, children in school, or families facing mortgage stress. The financial burdens and perceptions of livelihood risks associated with these expenses have a considerable negative impact on the mental health of urban residents (41). These major expenditures play a crucial role in moderating the development of economic equality among households, and the moderating effects of these variables are illustrated in Figure 1.

#### 2.2.5 Control variable

In this study, sociodemographic and socioeconomic factors are considered as control variables that may influence the development of family economic equality. The sociodemographic factors primarily include the age of the household head, marital status, family size, and household registration type. Socioeconomic factors encompass medical insurance (medsure), child dependency ratio (chideprat), older adult dependency ratio (olddeprat), and GDP per capita. Specifically, health insurance is coded as 1 for those who have purchased it and 0 for those who have not. Marital status is assigned a value of 1 for married individuals and 0 for others. Additionally, the nature of the account is coded as 1 for urban accounts and 0 for rural accounts. Age, family size, child dependency ratio, older adult dependency ratio, and per capita GDP are treated as continuous variables. Table 2 provides detailed definitions, coding schemes, and descriptive statistics for these key variables.

**Table 2:** Descriptive statics of the variables.

Variable	Obs	Mean	SD	Min	Max
Economic equality development	18,141	0.236	0.187	0.001	0.781
Social welfare	18,141	25.585	1.693	21.749	29.444
Emp_Qual	4,497	0.393	0.350	0.004	0.997
HC_Exp	15,819	6.583	2.505	0.000	10.597
Edu_Exp	16,520	3.474	4.004	0.000	10.158
Hou_Exp	14,649	8.005	0.684	5.866	10.297
Age	18,141	51.097	13.766	16.000	95.000
Medsure	18,141	0.933	0.250	0.000	1.000
Marriage	18,141	0.957	0.203	0.000	1.000
Hukou	18,141	0.320	0.466	0.000	1.000
Familysize	18,141	3.815	1.874	1.000	19.000
Chideprat	18,141	0.164	0.181	0.000	1.000
Olddeprat	18,141	0.170	0.293	0.000	1.000
lngdp	18,141	10.901	0.419	10.218	12.009

### 2.3 Model setting

This paper employs a bi-directional fixed-effect panel model to examine the influence of government social welfare expenditure on the development of family economic equality. I adopt a bidirectional fixed-effect model, which effectively controls for unobserved individual and time effects, thereby reducing bias caused by omitted variables. The primary advantage of this model is its ability to capture heterogeneity within the sample while accounting for time variations and individual differences. However, a notable limitation of fixed-effect models is that small sample sizes can lead to instability in model estimates. Consequently, I emphasize the importance of sample size during the model setup process to ensure the robustness of the estimates. The model is structured as follows:


(1) $$eco_{it}=\alpha_{0}+\alpha_{1}socialwelfare_{it}+\alpha_{2}X_{it}+\delta_{t}+\theta_{i}+\varepsilon_{it}$$

In Equation (1), ‘*eco_it_*’ denotes the explained variable, which represents the equitable development of the family economy. The variable ‘*socialwelfare_it_*’ serves as the primary explanatory variable, reflecting the government’s expenditure on social welfare. Meanwhile, ‘*X_it_*’ encompasses the control variables, which include both sociodemographic and socioeconomic factors. The terms ‘*δ_t_*’ and ‘*θ_i_*’ indicate the year fixed effect and individual fixed effect, respectively, while ‘*ε_it_*’ represents a random disturbance term.

Additionally, in order to address the limitations of the traditional three-step mediation effect model, the mechanism analysis section focuses solely on regressing the core explanatory variable ‘*socialwelfare_it_*’ on the mechanism variable ‘*M_it_*’. The impact of ‘*M_it_*’ on the explained variable ‘*eco_it_*’ will be examined through theoretical elaboration and literature support (42). The proposed model is outlined below:


(2) $$M_{it}=\gamma_{0}+\gamma_{1}socialwelfare_{it}+\gamma_{2}X_{it}+\delta_{t}+\theta_{i}+\varepsilon_{it}$$

In Equation (2), ‘*M_it_*’ represents the mechanism variable and denotes the employment quality of the household head, while $\gamma_{0}$ , $\gamma_{1}$ , and $\gamma_{2}$ are coefficients that need to be estimated. The core explanatory variable, ‘*socialwelfare_it_*’, along with the control variable, ‘*X_it_*’, is consistent with model (1).

In the section on variable analysis, this paper employs the moderate effect model to examine the influence mechanism of social welfare expenditure on the development of family economic equality. The specific model is outlined as follows:


(3) $$\begin{array}{l} eco_{it}=\beta_{1}+\beta_{2}socialwelfare_{it}+\beta_{3}W_{it}+\\ \beta_{4}socialwelfare_{it}\times W_{it}+\beta_{5}X_{it}+\varepsilon_{it} \end{array}$$

In Equation (3), ‘*eco_it_*’ represents the equitable development of the family economy, ‘*socialwelfare_it_*’ represents the social welfare expenditure, ‘*W_it_*’ is the moderating variable, ‘*X_it_*’ is the control variable, $\beta_{4}$ represents the moderating effect of the moderating variable on the social welfare expenditure and the development of family economic equality, and ‘*ε_it_*’ is the random disturbance term.

## 3 Results

### 3.1 Baseline regression

The two-way fixed effect model is employed to estimate the impact of social welfare expenditure on the development of family economic equality. Model 1 controls only for the core explanatory variables, as well as individual and year fixed effects, while Model 2 incorporates a series of additional control variables based on Model 1. As illustrated in Table 3, the coefficient for social welfare expenditure is significantly positive at the 1% statistical level, regardless of whether only core explanatory variables are controlled or additional control variables are included. Specifically, there exists a significant positive relationship between social welfare expenditure and the equitable development of family economics. A one percentage point increase in social welfare expenditure corresponds to an increase of 0.033 in the level of family economic equality development.

**Table 3:** Fixed effects of social welfare expenditure and economic equality development.

Variable	Economic equality development	
	Model 1	Model 2
Social welfare	0.032***	0.033***
	(4.071)	(4.289)
Age	–	−0.002
	–	(−0.454)
Medsure	–	0.005
	–	(0.831)
Marriage	–	−0.027*
	–	(−1.672)
Hukou	–	0.036***
	–	(3.817)
Familysize	–	0.005***
	–	(2.588)
Chideprat	–	−0.001
	–	(−0.037)
Olddeprat	–	−0.014
	–	(−1.290)
Lngdp	–	−0.037
	–	(−0.578)
_cons	−0.589***	−0.129
	(−2.957)	(−0.170)
Individual	Yes	Yes
Year	Yes	Yes
N	18,141	18,141
R^2^	0.042	0.048
Within R^2^	0.042	0.048
F	66.333	21.263

Levels of statistical significance (***p < 0.01, **p < 0.05, *p < 0.1).

### 3.2 Endogenetic analysis

The regression results presented above indicate a significant correlation between social welfare expenditure and the development of family economic equality; however, potential endogeneity issues within the model cannot be dismissed. This paper employs a two-stage least squares estimation (2SLS), utilizing the one-period lag of social welfare expenditure as an instrumental variable. The estimated results are summarized in Table 4. The first-stage estimation results demonstrate that the one-period lag of social welfare expenditure is significant at the 1% level, with an F-statistic for the instrumental variables of 3196.154 (*F* value > 10). This finding suggests that the chosen instrumental variables do not suffer from weak instrument problems. Furthermore, the results from the second-stage estimation reveal that social welfare expenditure remains significant, with a positive coefficient at the 1% statistical level. This indicates that, after addressing the endogeneity concerns, social welfare expenditure continues to exert a positive influence on the development of family economic equality.

**Table 4:** Regression of instrumental variables of social welfare expenditure to economic equality development.

Variable	First	Second
	Model 1	Model 2
Social welfare	–	0.513***
	–	(3.381)
l. Social welfare	0.146***	–
	(16.856)	–
Age	0.139***	−0.005
	(35.217)	(−0.984)
Medsure	−0.027**	0.012
	(−2.022)	(1.264)
Marriage	−0.001	−0.005
	(−0.021)	(−0.223)
Hukou	0.018	0.034***
	(0.985)	(2.668)
Familysize	−0.002	0.008***
	(−0.591)	(3.147)
Chideprat	−0.046	0.007
	(−1.262)	(0.287)
Olddeprat	−0.007	−0.008
	(−0.276)	(−0.442)
lngdp	1.056***	−0.229*
	(19.585)	(−1.671)
_cons	3.342***	−10.025**
	(8.422)	(−2.431)
Individual	Yes	Yes
Year	Yes	Yes
N	18,141	18,141
R^2^	0.844	
Within R^2^	0.844	
F	3196.154	

Levels of statistical significance (***p < 0.01, **p < 0.05, *p < 0.1).

### 3.3 Robustness test

To ensure the reliability of the baseline regression results, this paper employs three primary methods to test robustness. First, the core explanatory variables are substituted with expenditures from the unemployment insurance fund and the social pension insurance fund for urban and rural residents to measure social welfare expenditure. Second, samples from municipalities directly under the central government are excluded, and a two-way fixed effect regression is conducted to mitigate the influence of the unique characteristics and policy biases associated with these municipalities. Finally, all continuous variables undergo a 2% tail reduction to diminish the impact of extreme values on the regression outcomes, followed by re-estimation. The results of the robustness tests are presented in Table 5. Social welfare expenditure consistently shows a positive relationship in all cases and is statistically significant at the 1% level, indicating that higher government social welfare expenditure correlates with an increased level of economic equality development within families. This conclusion aligns closely with the previous estimation results, further confirming the robustness of the empirical regression findings in this paper. Overall, these regression results support hypothesis 1.

**Table 5:** Robustness test of social welfare expenditure and economic equality development.

Variable	Economic equality development		
	Model 1	Model 2	Model 3
Social welfare	0.038***	0.036***	0.033***
	(6.978)	(3.988)	(4.304)
Age	−0.002	−0.001	0.001
	(−0.381)	(−0.348)	(0.347)
Medsure	0.004	0.003	0.005
	(0.629)	(0.469)	(0.819)
Marriage	−0.027*	−0.016	−0.027*
	(−1.686)	(−1.037)	(−1.663)
Hukou	0.035***	0.042***	0.036***
	(3.741)	(4.146)	(3.811)
Familysize	0.005**	0.004**	0.005***
	(2.565)	(2.295)	(2.591)
Chideprat	0.000	0.001	−0.001
	(0.025)	(0.052)	(−0.079)
Olddeprat	−0.013	−0.011	−0.015
	(−1.162)	(−0.858)	(−1.373)
lngdp	−0.099	−0.294***	−0.039
	(−1.351)	(−5.712)	(−0.613)
_cons	0.718	2.484***	−0.254
	(0.909)	(3.618)	(−0.340)
Individual	Yes	Yes	Yes
Year	Yes	Yes	Yes
N	18,141	16,300	18,141
R^2^	0.054	0.067	0.048
Within R^2^	0.054	0.067	0.048
F	21.559	29.049	21.447

Levels of statistical significance (***p < 0.01, **p < 0.05, *p < 0.1).

### 3.4 Mediation effect analysis

Based on relevant literature and theoretical derivation, this study hypothesizes that social welfare expenditure can influence the equitable development of family economies by impacting the quality of employment, a proposition that will be examined in the following paragraphs. Model 1 in Table 6 presents the regression results regarding employment quality (Emp_Qual) in relation to social welfare expenditure. The findings reveal a significantly negative coefficient for social welfare expenditure on employment quality, indicating that a 1% increase in social welfare expenditure corresponds to a decrease of 0.069 in the employment quality of household heads. This suggests that increased social welfare expenditure does not promote high-quality employment. Several factors may contribute to this outcome. Firstly, there may be issues related to the implementation of social welfare policies or inefficiencies in their execution; thus, government social welfare expenditure may not effectively translate into actual employment-enhancing measures. For instance, funds may be allocated to short-term welfare payments rather than long-term job creation or training programs aimed at enhancing employability skills. Secondly, social welfare expenditure may predominantly benefit low-skilled workers, while high-quality jobs typically demand higher levels of skills and education. Additionally, the sample distribution may be skewed due to the high proportion of rural household registrations; among the 4,497 samples analyzed for employment quality, 2,474 are from agricultural households and 2,023 from non-agricultural (urban) households. Many migrant workers often lack formal labor contracts with their employers, resulting in poor working conditions where long hours are commonplace. Similarly, urban household heads also experience suboptimal working environments and employment quality. Due to the increased pressures of the employment environment and the high cost of living for families, many skilled workers are compelled to lower their employment standards. They often find themselves compromising with unequal work platforms or units, ultimately accepting low-quality job opportunities and conditions. In summary, it is an undeniable reality that the employment quality of Chinese household heads remains low (see Table 2), and the regression results corroborate this reality, aligning with hypothesis 2.

**Table 6:** Mediating effect of social welfare expenditure and economic equality development.

Variable	Emp_Qual	
	Model 1	Model 2
Social welfare	−0.069**	−0.069*
	(−1.968)	(−1.857)
Age		0.041
		(1.045)
Medsure		0.025
		(0.727)
Marriage		0.060
		(0.735)
Hukou		0.011
		(0.193)
Familysize		0.005
		(0.435)
Chideprat		−0.092
		(−0.883)
Olddeprat		−0.104
		(−1.116)
lngdp		−0.089
		(−0.600)
_cons	2.104**	1.318
	(2.357)	(0.486)
Individual	Yes	Yes
Year	Yes	Yes
N	4,497	4,497
R^2^	0.034	0.040
Within R^2^	0.034	0.040
F	9.734	3.009

Levels of statistical significance (***p < 0.01, **p < 0.05, *p < 0.1).

### 3.5 Moderating effect analysis

Table 7 presents the estimated moderating effects of family healthcare expenditure (HC_Exp), family education expenditure (Edu_Exp), and family housing expenditure (Hou_Exp) on government social welfare expenditure, with the objective of enhancing economic equality among families. In Model 1, the interaction term between family healthcare expenditure and social welfare expenditure is significantly negative, suggesting that family healthcare expenditure has a notable moderating effect on the relationship between social welfare expenditure and the development of family economic equality. Specifically, when family healthcare expenditure is high, the positive influence of government social welfare expenditure on the advancement of family economic equality diminishes. This phenomenon may arise because elevated healthcare spending increases the financial burden on households, thereby partially counteracting the beneficial impact of government social welfare expenditure. This also indirectly highlights the shortcomings of existing healthcare reforms and the overall healthcare system. In Model 2, the interaction between family education expenditure and social welfare expenditure is not significant, indicating that the moderating effect of family education expenditure on the relationship between social welfare expenditure and family economic equality is minimal. Similarly, in Model 3, the interaction between household housing expenditure and social welfare expenditure is not significant, suggesting that the moderating effect of household housing expenditure on the relationship between social welfare expenditure and the development of household economic equality is also negligible.

**Table 7:** Moderating effect of social welfare expenditure and economic equality development.

Variable	(1)	(2)	(3)
	Model 1	Model 2	Model 3
Social welfare	0.032***	0.034***	0.033***
	(3.610)	(3.999)	(4.090)
HC_Exp	−0.002**	–	–
	(−2.227)	–	–
Social welfare×HC_Exp	−0.001*	–	–
	(−1.896)	–	–
Edu_Exp	–	0.000	–
	–	(0.227)	–
Social welfare×Edu_Exp	–	0.000	–
	–	(1.120)	–
Hou_Exp	–	–	0.005
	–	–	(1.399)
Social welfare×Hou_Exp	–	–	0.001
	–	–	(0.745)
Age	0.005	−0.004	0.002
	(0.916)	(−0.552)	(0.461)
Medsure	0.004	0.008	0.014*
	(0.523)	(1.219)	(1.960)
Marriage	−0.032	−0.029*	−0.042***
	(−1.600)	(−1.787)	(−2.655)
Hukou	0.032***	0.033***	0.038***
	(3.191)	(3.240)	(3.387)
Familysize	0.006***	0.006***	0.004
	(2.671)	(2.804)	(1.638)
Chideprat	0.007	0.002	0.003
	(0.354)	(0.082)	(0.176)
Olddeprat	−0.012	−0.010	−0.015
	(−0.936)	(−0.884)	(−1.113)
lngdp	−0.032	−0.040	−0.131
	(−0.453)	(−0.565)	(−1.453)
_cons	−0.489	−0.012	0.692
	(−0.575)	(−0.014)	(0.650)
Individual	Yes	Yes	Yes
Year	Yes	Yes	Yes
N	15,819	16,520	14,649
R^2^	0.056	0.048	0.047
Within R^2^	0.056	0.048	0.047
F	16.953	15.232	13.379

Levels of statistical significance (***p < 0.01, **p < 0.05, *p < 0.1).

Family healthcare expenditure undermines the positive impact of social welfare expenditure on the development of economic equality within families, primarily for the following reasons: First, the commercialization and privatization of medical resources significantly affect this dynamic. The commercial operation of medical resources inevitably leads to a rapid increase in medical costs. To ensure survival and growth, hospitals and enterprises have adopted business models such as “supporting medicine with illness” or “supporting medicine with medicine, “resulting in these costs ultimately being transferred to the general population. Second, inadequate medical insurance reimbursement coverage and rates further exacerbate the situation. With the growing challenges posed by an aging population, food safety concerns (such as the misuse of antibiotics, additives, and preservatives), life stress, chronic diseases, and various sudden or infectious diseases, many medical conditions are frequently not covered by insurance. Consequently, individuals are often required to bear substantial or full expenses to alleviate their pain and suffering, thereby directly increasing their economic burden. Additionally, many medical insurance plans are only valid within urban areas, and the procedures for cross-city medical treatment can be exceedingly cumbersome. The significant variability in local medical insurance policies further contributes to high medical costs for ordinary individuals. The privatization and commercialization of medical resources can lead to patients being viewed as instruments for profit by companies, such as hospitals and pharmacies. This concern is one of the key reasons many countries advocate for universal free healthcare. The aforementioned issues may significantly contribute to the limited actual benefits of family healthcare, thereby causing family healthcare expenditures to notably hinder the positive effects of government spending on public welfare and family economic equality. In summary, this regression result partially validates the existence of Hypothesis 3.

### 3.6 Heterogeneity analysis

This paper further examines the regional and urban–rural heterogeneity. On one hand, variations in economic levels, cultural backgrounds, policy environments, and other factors across different regions in China may influence both the manner and extent of social welfare expenditure aimed at promoting equitable development of family economies. On the other hand, significant disparities in resource allocation between urban and rural areas in education, healthcare, housing, and other domains will impact the effectiveness of social welfare expenditure in fostering equitable development of family economies.

Through a comparative analysis of the impact of social welfare expenditure on the development of family economic equality across eastern, central, and western regions, as well as urban and rural areas, we identified several significant differences, as illustrated in Model 1, Model 2, and Model 3 in Table 8. Social welfare expenditure has a notably positive impact on family economic equality in eastern China. Conversely, in the central region, there is a significant negative effect, while in the western region, the impact is not significant. This disparity can be attributed to the relatively developed economy and comprehensive public services in the eastern region, where government social welfare expenditures can generate scale and agglomeration effects, effectively promoting equitable development of family economies. In contrast, the economies of the central and western regions are relatively underdeveloped, with inadequate public services and infrastructure. Consequently, the effect of social welfare expenditure on the equitable development of family economies is either insignificant or even negative. In the central region, issues such as weak policy implementation and opaque fund utilization may hinder the effectiveness of social welfare expenditures. In the western region, the promotion of social welfare expenditure projects may be constrained by objective environmental factors, such as geography and resource availability, leading to challenges in project implementation and negligible effects.

**Table 8:** Heterogeneity analysis of social welfare expenditure and economic equality development.

Variable	Economic equality development				
	East	Central	West	Rural	Urban
	Model 1	Model 2	Model 3	Model 4	Model 5
Social welfare	0.051***	−0.083***	0.017	0.061***	−0.007
	(6.635)	(−5.948)	(0.547)	(5.279)	(−1.009)
Age	−0.007	0.007	0.001	−0.002	−0.002
	(−1.633)	(1.147)	(0.199)	(−0.348)	(−0.299)
Medsure	0.013*	−0.005	−0.019	−0.001	0.003
	(1.843)	(−0.364)	(−1.147)	(−0.132)	(0.328)
Marriage	−0.016	−0.007	−0.053	0.002	−0.020
	(−0.958)	(−0.238)	(−1.395)	(0.132)	(−0.862)
Hukou	0.024**	0.039**	0.049**	–	–
	(2.115)	(2.461)	(2.163)	–	–
Familysize	−0.000	0.004	0.010***	0.006***	0.003
	(−0.051)	(1.233)	(3.284)	(2.609)	(0.811)
Chideprat	0.035*	0.014	−0.035	0.006	−0.017
	(1.886)	(0.431)	(−0.987)	(0.304)	(−0.712)
Olddeprat	−0.012	−0.022	0.016	−0.024	0.001
	(−1.033)	(−0.803)	(0.546)	(−1.455)	(0.055)
lngdp	0.124	−0.997***	−0.729***	−0.075	0.002
	(1.040)	(−9.176)	(−6.593)	(−0.970)	(0.027)
_cons	−2.085	12.528***	7.362***	−0.514	0.617
	(−1.516)	(8.773)	(6.274)	(−0.561)	(0.585)
Individual	Yes	Yes	Yes	Yes	Yes
Year	Yes	Yes	Yes	Yes	Yes
N	8,819	4,424	4,898	12,337	5,804
R^2^	0.066	0.277	0.122	0.085	0.075
Within R^2^	0.066	0.277	0.122	0.085	0.075
F	18.649	43.372	16.286	25.104	19.151

Levels of statistical significance (***p < 0.01, **p < 0.05, *p < 0.1).

As demonstrated in Model 4 and Model 5 in Table 8, social welfare expenditure significantly enhances the development of family economic equality in rural areas, while this effect is not observed in urban areas. This discrepancy can be attributed to the relatively lower pressures faced by rural households regarding education, healthcare, housing, and food, as well as the generally lower cost of living compared to urban settings. Consequently, the positive influence of government social welfare expenditure on the economic equality of families in rural areas is fully realized. In contrast, in urban areas, the high cost of living may counterbalance the positive effects of government social welfare expenditure on the development of economic equality among households. In summary, this variation underscores the need for the government to optimize, upgrade, and adjust social welfare expenditure in accordance with specific local conditions, thereby enabling the introduction of more targeted and effective measures to enhance the overall economic equality of families across China.

## 4 Discussion and limitations

### 4.1 Discussion and implication of the findings

The results of this study reinforce the significance of considering social welfare expenditure as a crucial determinant of equitable economic development. Through an empirical analysis of CFPS data, our findings reveal a strong correlation between social welfare expenditure and the advancement of economic equality, thereby underscoring the necessity for comprehensive social and economic interventions in this domain. For many individuals in China, more substantial social welfare measures can effectively alleviate poverty (9, 10), thereby addressing the disparity between the rich and the poor (8). Our results indicate that for every 1% increase in social welfare expenditure, the level of family economic equality development increases by 0.033, confirming the direct impact of social welfare expenditure on the development of economic equality.

The mediation effect analysis indicates that for every 1% increase in social welfare expenditure, the employment quality of the head of household decreases by 0.069. This suggests that social welfare expenditure negatively impacts the employment quality of the head of household, which in turn affects the level of equitable economic development within families. This phenomenon may be attributed to the lag in social welfare policies or the insufficient protection, supervision, and rights enforcement for temporary ‘migrant workers’ or low-skilled positions, as outlined by the ‘Labor Security Law’. Consequently, the current social welfare expenditure fails to effectively enhance the employment quality of the head of household, thereby hindering the equitable economic development of families.

The interaction term between family healthcare expenditure and social welfare expenditure is significantly negative, indicating that family healthcare expenditure has a considerable negative regulatory effect on the relationship between social welfare expenditure and the development of family economic equality. This suggests that high medical care expenditure increases the economic burden on families, partially offsetting the positive impact of government social welfare expenditure, and indirectly reflecting the failures and inadequacies of the current medical reform and healthcare system (20–22). Furthermore, the impact of social welfare expenditure on economic equality development varies across different regions. In contrast, due to the relatively developed economy and comprehensive public services in the eastern region, government social welfare expenditure can create a scale and agglomeration effect, effectively promoting the equitable development of family economies. In comparison to urban areas, rural regions experience less pressure regarding education, healthcare, housing, and food, coupled with lower living costs, allowing the positive role of government social welfare expenditure in promoting the equitable development of family economies to be fully realized.

### 4.2 Policy suggestions for improving social welfare expenditure

Social welfare expenditure is essential for ensuring the basic livelihoods of citizens and for the functioning of the national social security system. It plays a critical role in promoting social equity, justice, and sustainable development. Such expenditure must be scientific, efficient, and targeted, without compromising the quality of jobs or health services. Enhancing the quality of employment and reforming healthcare are vital measures for achieving economic equality and improving family well-being.

On one hand, the government should prioritize enhancing the employment quality of residents by establishing and refining a social welfare security system focused on employment quality. Employment serves as the fundamental livelihood for individuals, and families without employment risk losing their financial resources, potentially leading to poverty and exacerbating economic inequality within households. First, the government should provide unemployed individuals and families with a specific amount of unemployment compensation, determined by the local economic development level, to ensure their basic living needs are met (43). For low-income families, particularly those with children, older adult members, or spouses who have lost their income, unemployment benefits should be increased to double the standard amount, thereby promoting economic equality among families. Second, local governments must concentrate on increasing job opportunities, expanding employment, and enhancing employment quality while actively addressing the issue of structural unemployment. This can be accomplished by improving the public employment service system to support key demographics, such as recent graduates and disadvantaged groups. Additionally, a lifelong vocational skills training system should be implemented to continuously enhance employment skills and levels, enabling disadvantaged groups to access greater development opportunities and achieve economic equality (44). In summary, the government must ensure that at least one member of every family is employed. Third, the government should improve and guide the entrepreneurship support system to stimulate job creation, standardize the development of new employment forms, and promote high-quality employment. For enterprises, companies, or organizations that create new jobs, the government should offer specific subsidies or tax incentives. A high level of employment quality is a crucial factor in optimizing the structure of government social welfare expenditures and ensuring equitable development of the family economy.

On the other hand, the government should implement reforms to the medical system and promote a “free medical system” (45, 46). In China, although many individuals purchase health insurance, these policies often do not cover all costs when they become ill, typically covering only a significant portion (about 60%). Consequently, it is common for many people to incur excessive medical expenses or to fall into poverty due to illness, which can be attributed to several factors. First, the rapid rise in medical costs has a substantial impact (47). Despite the increasing percentage of expenses reimbursed by state health insurance each year, families’ healthcare expenditures have not decreased. For instance, previously, if a person contracted a cold, the total cost would be 100 yuan, with a reimbursement ratio of 50%; thus, the out-of-pocket payment would be 50 yuan. However, if someone catches a cold now, the total cost is 1,000 yuan, and although the reimbursement rate has risen to 80%, the self-payment amounts to 200 yuan. While the reimbursement proportion has improved from 50 percent to 80 percent, the out-of-pocket expenses for ordinary individuals have increased from 50 yuan to 200 yuan, representing a four-fold rise. More critically, in practice, the average Medicare reimbursement rate is often less than 80%, typically ranging between 50 and 70%. This indicates that individuals must bear a significant amount of out-of-pocket costs. Therefore, progress in healthcare reform should not solely focus on reimbursement rates; it must also consider the actual amount individuals pay for treatment and whether these amounts are lower than in the past. Second, we examine the impact of payment methods on medical expenses. For patients, regardless of the type of disease, the prevailing procedure for conditions that necessitate hospitalization typically follows the principle of “pay first - see a doctor later - then reimburse” (48). This payment structure imposes significant financial strain on many patients from the outset of their medical journey. Additionally, it creates an operational environment that may enable hospitals to engage in “excessive medical treatment, “generating substantial profits. In China, where many hospitals operate as private or for-profit enterprises, the need to survive and generate revenue often compels these institutions to resort to practices such as “excessive medical treatment” or the reliance on “medicine to support medicine.” This often leads to a dependence on funds reimbursed by patients and the government. Even public hospitals frequently rely on income from drug sales and diagnostic fees to remain viable. Consequently, hospitals may exhibit a tendency to prescribe costly medications and tests during treatment, further exacerbating the financial burden on patients. Moreover, due to the complexities of medical knowledge and technical barriers, many patients find themselves in a vulnerable position, akin to “lambs to the slaughter.” When confronted with the urgency of preserving life, financial concerns often become inconsequential. Third, inadequate health insurance also plays a significant role. Although China has established a basic medical insurance system, many patients continue to face the issue of underinsurance. Numerous expensive drugs and specialized treatments are not covered by insurance, forcing patients to shoulder the high costs of their treatment (49). Even with insurance, reimbursement rates often fail to cover the full expenses, leaving patients to bear the financial burden. Over the years, despite substantial state investment in the medical sector, the persistent approach of prioritizing profit over patient care—characterized by the model of ‘treating patients with diseases’—and the lack of reform toward ‘free medical care for all’ hinder the resolution of healthcare accessibility and the issue of exorbitant medical costs. In today’s society, it is noteworthy that even North Korea, often viewed as backward, has achieved ‘free medical care for all, ‘while China, as the world’s second-largest economy, has yet to realize this goal, highlighting a significant irony and a cause for shame.

The government should implement healthcare system reforms and promote a universal free healthcare policy (45, 46). Specifically, the Chinese government ought to undertake comprehensive healthcare policy reforms and advocate for universal free medical care, returning the healthcare sector to its fundamental public welfare orientation. A growing body of empirical evidence supports the feasibility of implementing universal free healthcare in China (45, 46, 50, 51).

For example, countries such as India, Cuba, North Korea, Pakistan, and Tanzania—all of which have significantly lower per capita GDP than China—have already established systems of universal free healthcare. Cuba, in particular, not only provides free healthcare to all its citizens but is also known for offering high-quality medical services. In 2021, Cuba’s per capita GDP was $7,291, while China’s was approximately $12,500—substantially higher than Cuba’s during the same period. If Cuba can achieve high-quality universal healthcare, why has China—despite its economic advantage—yet to do so? The persistent issues of expensive and inaccessible medical care in China are deeply concerning. Shouldn’t this be considered a national shortcoming?

Take North Korea as another example: in 2024, China’s estimated per capita GDP is about $13,445, while North Korea’s is reportedly around $2,000—a mere fraction of China’s. Yet North Korea has managed to implement a universal free healthcare system. If countries with far fewer resources can do it, why cannot China? Some may argue that although countries like North Korea provide free healthcare, their medical conditions are poor or limited. However, does the absence of free healthcare—characterized instead by high costs or excessive treatment—necessarily lead to better healthcare conditions? Does it guarantee unlimited access to resources? The answer is clearly no.

In conclusion, China possesses both the economic capacity and structural conditions necessary to achieve universal free healthcare, particularly in comparison to other developing nations such as Cuba, India, and North Korea. The proposed policy is both economically feasible and practically implementable. Specifically, the following measures are recommended: First, waive the annual medical insurance premium of approximately 400 RMB for all citizens to ensure access to basic healthcare services for everyone, regardless of their insurance status. Second, substantially increase the medical reimbursement rate for the general population, gradually progressing toward full coverage. Third, implement a ‘treatment first, payment later’ policy to genuinely safeguard citizens’ rights to health and life. These reforms would alleviate the burden of high medical costs, protect public health, enhance social welfare, and ultimately contribute to greater economic equality at the household level.

### 4.3 Limitations

There are several limitations to this study that warrant further consideration. First, the measurement of social welfare expenditure is based on a consensus approach, primarily viewed from a national macroeconomic perspective. This approach does not account for important factors such as maternity leave benefits, childcare benefits, or temporary relief for pregnant women, which could serve as significant control variables. Secondly, the measurement method for economic equality development is limited. In this study, there is a scarcity of research specifically addressing “economic equality development” in China, with most references focusing on “common prosperity, “which emphasizes narrowing the income gap and enhancing overall wealth. To facilitate understanding, the relevant measurement indicators for “economic equality development” in this paper also draw from the measurement methods associated with “common prosperity.” Additionally, it is important to acknowledge the limitations in controlling for unobservable heterogeneity related to sociodemographic and socioeconomic factors. Finally, the micro-database analyzed primarily targets household-level data from 2016 to 2020, with the latest data for 2022 and 2024 not yet available. While the information gathered is relatively comprehensive, the temporal lag may restrict the timeliness and comprehensiveness of the research outcomes.

## 5 Conclusion

This study, based on the analysis of data from the Chinese Household Panel Study (CFPS), confirms the significant impact of social welfare expenditure on the development of economic equality. The results indicate a positive correlation between social welfare expenditure and the advancement of economic equality, revealing that a 1% increase in social welfare expenditure corresponds to a 0.033 increase in the level of economic equality development within households. This relationship is particularly evident in the eastern region and rural areas, underscoring the overall influence of social welfare expenditure on equitable economic development. However, intermediary effect analysis reveals that for each 1% increase in social welfare expenditure, the employment quality of household heads declines by 0.069. This suggests that social welfare expenditure does not effectively enhance the employment quality of household heads, thereby adversely affecting the economic equality of households. Additionally, moderating effect analysis indicates that the interaction between family healthcare expenditure and social welfare expenditure is significantly negative, suggesting that family healthcare expenditure mitigates the positive impact of social welfare expenditure on the development of family economic equality. Finally, heterogeneity analysis demonstrates that social welfare expenditure exerts a more pronounced effect on the development of household economic equality in eastern, central, and rural areas.

Based on the findings, the government should optimize and expand the level and efficiency of social welfare spending. For instance, the government should implement a policy of ‘free healthcare for all.’ Specifically, the annual medical insurance fee of approximately 400 yuan should be waived for all citizens to ensure that residents can access basic medical services, regardless of their ability to pay. Furthermore, the proportion of medical reimbursement for the general public should be significantly increased until full reimbursement is achieved. Additionally, the policy of ‘diagnosis and treatment first, settlement later’ should be implemented to effectively safeguard citizens’ rights to life and health. Moreover, the labor department should strengthen the protection of workers’ rights and interests, which includes establishing minimum monthly wage standards, setting maximum weekly working hours, and enforcing mandatory regulations such as labor contract signing to enhance the quality of employment. These measures will effectively ensure the basic needs of families, narrow the gap between the rich and the poor, and promote economic equity. Simultaneously, improving the precision and efficiency of social welfare spending will help increase household income and wealth accumulation while reducing the cost of living. This can be achieved through controlling housing prices, promoting the construction of low-rent housing, implementing free preschool education and 14 years of compulsory education, and providing at least 1,000 yuan per month in pensions for seniors over 60. Collectively, these measures will help optimize the level and efficiency of social welfare spending, promote equitable economic development, and further narrow the gap between the rich and the poor.

## Data Availability

Publicly available datasets were analyzed in this study. This data can be found at: https://cfpsdata.pku.edu.cn/#/home.

## Appendix

The economic equality development indicator is calculated as follows:

This study employs the entropy method to assess the level of economic equality development among households. By utilizing the entropy method, this research aims to eliminate the influence of subjective biases and provide an objective and accurate representation of the evaluation index’s contribution to the system. The calculation process involves the following steps: First, data standardization.

Positive indicators:

(1)
$$y_{\mathrm{ij}}^{t}=\frac{x_{\mathrm{ij}}^{t}-x_{\mathrm{jmin}}}{x_{\mathrm{jmax}-}x_{\mathrm{jmin}}}+0.0001$$

Negative indicator:

(2)
$$y_{\mathrm{ij}}^{t}=\frac{x_{\mathrm{jmax}-}x_{\mathrm{ij}}^{t}}{x_{\mathrm{jmax}-}x_{\mathrm{jmin}}}+0.0001$$

Where, $x_{\mathrm{ij}}^{t}$ is the jTH index of family i in year t.

Secondly, calculate the entropy of each index:

(3)
$$e_{j}=-k\overset{T}{\underset{t=1}{\sum }}\overset{m}{\underset{i=1}{\sum }}P_{\mathrm{ij}}^{t}\ln P_{\mathrm{ij}}^{t}$$

Where, $P_{\mathrm{ij}}^{t}=y_{\mathrm{ij}}^{t}/\overset{T}{\underset{t=1}{\sum }}\overset{m}{\underset{i=1}{\sum }}y_{\mathrm{ij}}^{t}$ , $k=\frac{1}{\ln\left(\mathrm{mT}\right)}$ , m is the number of samples and T is the number of years.

Thirdly, determine the weight of each indicator:

(4)
$$w_{j}=\left(1-e_{j}\right)/\overset{n}{\underset{j=1}{\sum }}\left(1-e_{j}\right)$$

Finally, the comprehensive score of each family was calculated:

(5)
$$S_{i}=\overset{n}{\underset{j=1}{\sum }}w_{j}y_{\mathrm{ij}}^{t}$$

The Kakwani relative deprivation index is a calculation method that offers the advantages of normality, dimensionlessness, and invariance of transfer. In this study, the Kakwani Index is utilized to assess both household income disparity and wealth disparity (household net worth). The specific calculation method involves sorting the different families xi in a total sample of n survey respondents X according to their income levels. The income vector X = (x1, x2,…, xn) is then used to calculate the value of income inequality per household, denoted as RD (xi, xj), through the following formula:

(6)
$$\mathrm{RD}\left(x_{i}{\mathrm{,x}}_{j}\right)=\frac{1}{n\mu_{x}}\overset{n}{\underset{j=i+1}{\sum }}\left(x_{j}-x_{i}\right)=\gamma_{x_{i}}^{+}\left[\left(\mu_{x_{i}}^{+}-x_{i}\right)/\mu_{x}\right]$$

Where, $\mu_{x_{i}}^{+}$ is the mean income (muxp) of the sample whose income exceeds family X in the total income vector $x_{i}$ ; $\gamma_{x_{i}}^{+}$ is the proportion of the number of samples whose income exceeds family $x_{j}$ in the total income vector X to X (gaxp); $\mu_{x}$ is the mean revenue (mux) of the total sample X. RD( $x_{i},x_{j}$ ) is between 0 and 1, and the closer the value is to 1, the lower the family’s relative income status in the group.
